# Supplementary material for: Characterization of gene expression changes over healthy term pregnancies
Source: PLoS One. 2018 Oct 10;13(10):e0204228. doi: 10.1371/journal.pone.0204228 (PMC6179206; doi:10.1371/journal.pone.0204228)
Supplement: S2 Table — (DOCX) [file pone.0204228.s002.docx]

**S2 Table:** Biological Processes associated with genes whose expression increases over pregnancy.

**
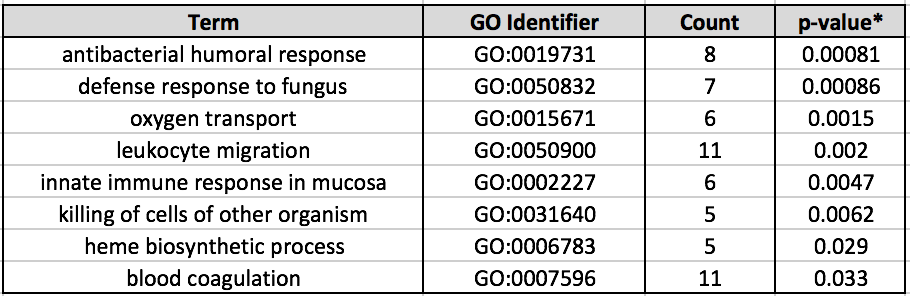
**
