# Supplementary figures and images for: Characterization of gene expression changes over healthy term pregnancies
Source: PLoS One. 2018 Oct 10;13(10):e0204228. doi: 10.1371/journal.pone.0204228 (PMC6179206; doi:10.1371/journal.pone.0204228)

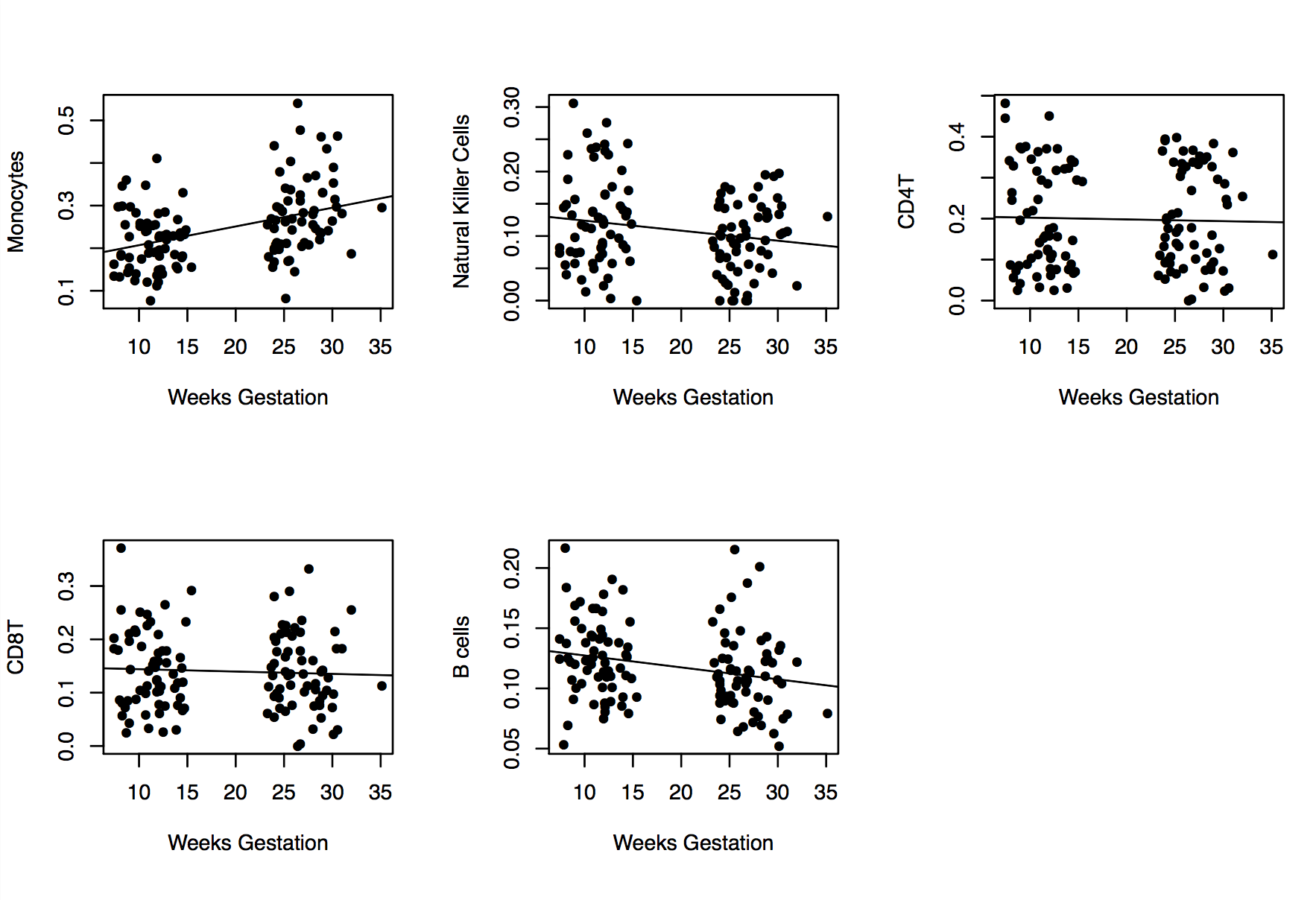

Supplement: S1 Fig — Monocytes increase over pregnancy, whereas B cells and natural killer cells decrease over pregnancy. Other evaluated cell types do not change. The x-axis represents the weeks of gestation at sample collection and the y-axis represents cell proportions. (PNG) [file pone.0204228.s003.png]

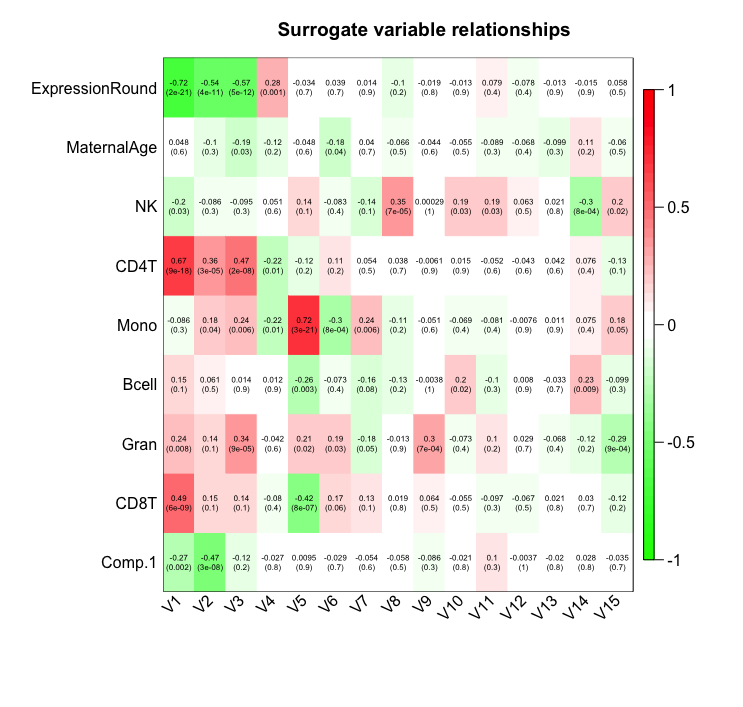

Supplement: S2 Fig — The grid consists of the correlation coefficient (r) for each pair on top with the p-value indicating significance of the correlation below. ExpressionRound indicates batch. Comp.1 indicates the first principal component for ancestry. Maternal age was measured in years. Cell composition (NK, CD4+ T cells, monocytes, B cells, granulocytes and CD8+ T cells) were estimated as described in the Methods. The intensity of the shading represents the correlation coefficient, with darker shading being associated with a higher correlation coefficient. Red shading represents a positive correlation coefficient and green shading represents a negative correlation coefficient. (PNG) [file pone.0204228.s004.png]

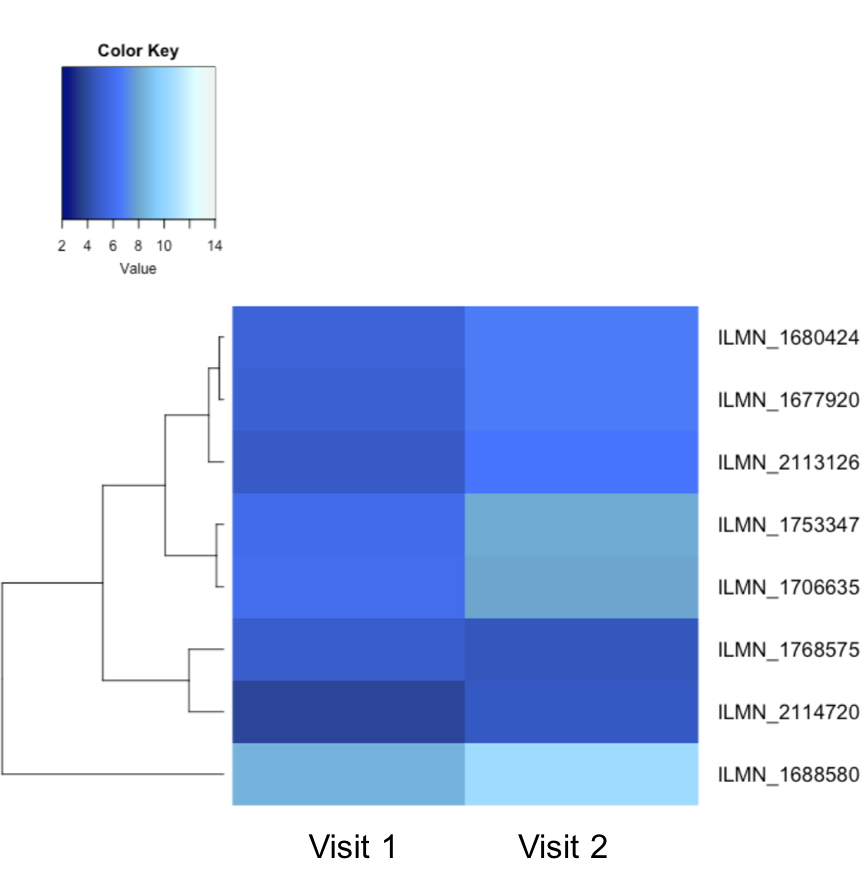

Supplement: S3 Fig — (PNG) [file pone.0204228.s005.png]

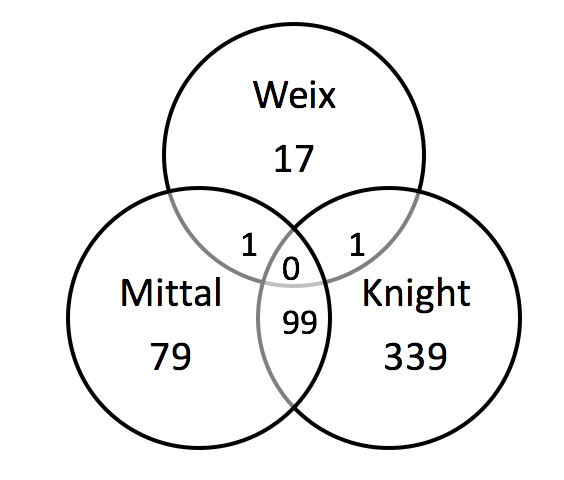

Supplement: S4 Fig — (PNG) [file pone.0204228.s006.png]
